# Laparoscopic-assisted natural orifice specimen extraction subtotal colectomy for redundant colon: a retrospective parallel-cohort comparative study with reduced surgical site infections and accelerated recovery

**DOI:** 10.3389/fsurg.2026.1926480

**Published:** 2026-09-10

**Authors:** Wen-Na Liu, Li-Hao Deng

**Affiliations:** 1 Department of Laboratory Medicine, Sichuan Provincial People’s Hospital, School of Medicine, University of Electronic Science and Technology of China, Chengdu, China; 2 Department of Gastrointestinal Hernia and Abdominal Wall Surgery, Affiliated Hospital of Chengdu University, Chengdu, Sichuan, China

**Keywords:** accelerated recovery, laparoscopic surgery, natural orifice specimen extraction, redundant colon, slow-transit constipation, subtotal colectomy, surgical wound infection

## Abstract

**Background:**

Redundant colon is one of the anatomical causes of slow transit constipation (STC). When conservative management fails, surgical intervention with subtotal colectomy is indicated. This study aimed to compare the perioperative outcomes of laparoscopy-assisted natural orifice specimen extraction (LA-NOSE) subtotal colectomy with conventional laparoscopy-assisted subtotal colectomy for redundant colon.

**Methods:**

A retrospective analysis was conducted on 34 patients with redundant colon who underwent laparoscopy-assisted subtotal colectomy with cecorectal anastomosis at the Affiliated Hospital of Chengdu University between January 2009 and January 2026. Of these, 16 patients underwent LA-NOSE and 18 underwent conventional surgery with a mini-laparotomy for specimen extraction. Categorical perioperative outcomes were compared using Fisher's exact test.

**Results:**

Baseline characteristics were comparable between the two groups. The LA-NOSE group showed certain advantages in postoperative recovery: sitting up within 24 h (87.5% vs. 38.9%, *p* = 0.005), getting out of bed within 2 days (93.8% vs. 55.6%, *p* = 0.019), ambulation within 3 days (100% vs. 72.2%, *p* = 0.046), return of gastrointestinal function within 2 days (62.5% vs. 27.8%, *p* = 0.045), liquid diet tolerance within 3 days (87.5% vs. 44.4%, *p* = 0.013), and earlier suture removal on days 6–7 (100% vs. 0%, *p* < 0.001). The incidence of surgical site infection (SSI) was significantly lower in the LA-NOSE group (0% vs. 27.8%, *p* = 0.046). Operative time, estimated blood loss, and the incidence of intestinal obstruction, anastomotic leakage, and intra-abdominal infection showed no significant differences between the groups.

**Conclusion:**

LA-NOSE subtotal colectomy is a safe and feasible minimally invasive option for redundant colon, offering accelerated postoperative recovery, reduced SSI, and superior cosmetic outcomes without compromising safety. This technique adheres to the principles of Enhanced Recovery After Surgery (ERAS) and offers an alternative treatment option for transnatural orifice specimen extraction in benign colonic diseases.

## Introduction

Chronic constipation (CC) is a highly prevalent gastrointestinal disorder that profoundly impairs quality of life (1). Slow-transit constipation (STC), as the rarest subtype of chronic constipation, is frequently associated with underlying colonic motility disorders, but is more commonly encountered in patients with refractory symptoms (2–5). Redundant colon is defined as an abnormally elongated and tortuous large bowel (6, 7), and it is one of the anatomical causes among the various etiologies of STC (8). Patients often present with intractable constipation, and surgical intervention becomes necessary when systematic conservative therapy fails for two or more years (9). Subtotal colectomy with cecorectal anastomosis (SCC-CRA), which preserves the ileocecal valve, has been shown to effectively relieve symptoms while reducing the incidence of debilitating postoperative diarrhea by maintaining intestinal barrier and absorptive functions (10–12).

Conventionally, laparoscopy-assisted subtotal colectomy requires an abdominal mini-laparotomy to extract the resected specimen. This auxiliary incision represents one of the major sources of postoperative somatic pain, surgical site infection (SSI), incisional hernia, and delayed mobilization, and may partially attenuate the benefits of the minimally invasive approach (13–19). In recent years, Natural Orifice Specimen Extraction Surgery (NOSES) emerged as a technique to explore more minimally invasive approaches for abdominal surgery, with the specimen retrieved transanally or transvaginally, thereby eliminating the need for an abdominal extraction incision (20, 21). Laparoscopy-assisted NOSES (LA-NOSE) applies this principle to colon resections, potentially offering superior postoperative recovery, reduced wound-related morbidity, and a “scarless” abdomen (22).

However, data specifically examining LA-NOSE for subtotal colectomy in benign STC due to redundant colon remain sparse (23, 24). This retrospective study was designed to compare the perioperative outcomes, with a primary focus on the incidence of surgical site infection (SSI) and postoperative recovery milestones (including mobilization and return of gastrointestinal function), between LA-NOSE and conventional laparoscopy-assisted subtotal colectomy in patients with redundant colon. Secondary outcomes included operative time, estimated blood loss, and other complication rates.

## Patients and methods

### Study design and patients

This is a retrospective, parallel-cohort comparative study. Based on the surgical period and the technique used for specimen extraction, patients were divided into two groups: conventional laparoscopy-assisted subtotal colectomy and LA-NOSE subtotal colectomy. A subset of patients in the conventional surgery group (*n* = 10) were previously reported in a technical feasibility study that focused on the description of surgical procedures. The present study represents a substantial expansion of that earlier work, incorporating a parallel-cohort comparative design, an enlarged sample size (*n* = 34), systematic comparisons of outcome measures, and a specific focus on the clinical advantages of LA-NOSE over conventional laparoscopic surgery. The two articles do not overlap in terms of research questions, analytical approaches, or reported outcomes. The study design and reporting follow the STROBE (Strengthening the Reporting of Observational Studies in Epidemiology) checklist for observational studies. All patients underwent standardized objective assessments prior to surgery, including colonic transit study (marker method), defecography, anorectal manometry, balloon expulsion test, pelvic floor electromyography, and endoanal ultrasound, to confirm the diagnosis of isolated slow-transit constipation and exclude outlet obstruction or pelvic floor dysfunction. The study protocol was approved by the Institutional Review Board, and written informed consent was obtained from all patients.

Inclusion criteria:

1. STC meeting Rome IV criteria (25), excluding outlet obstruction or mixed constipation
2. Barium enema confirming redundant colon
3. Failure of ≥2 years of systematic non-surgical treatment
4. Severe impact on daily life and strong surgical willingness
5. Prolonged colonic transit time, excluding outlet obstruction

Exclusion criteria:

1. Secondary constipation (organic, drug-induced, or IBS-related)
2. Emergency surgery for colonic torsion or obstruction
3. Prior abdominal or pelvic surgery
4. Incomplete clinical data or loss to follow-up

### Perioperative management

Throughout the entire study period (2009–2026), all patients in both groups received a standardized perioperative care protocol based on the Enhanced Recovery After Surgery (ERAS) principles for elective colorectal surgery. The ERAS protocol comprised the following key elements across the preoperative, intraoperative, and postoperative phases:

Preoperative phase: All patients received standardized preadmission education and counseling regarding the surgical procedure, expected postoperative course, and recovery milestones. Preoperative risk assessment and optimization of comorbidities were performed according to a uniform protocol. Mechanical bowel preparation was administered to all patients using oral polyethylene glycol electrolyte powder starting at 5:00 PM on the day before surgery, continuing until clear, residue-free effluent was achieved. Preoperative fasting was standardized, with clear fluids permitted up to 2 h before surgery. No routine preoperative sedative premedication was given.

Intraoperative phase: All procedures were performed under standardized general anesthesia combined with thoracic epidural analgesia, as recommended by ERAS guidelines. Intraoperative fluid management followed a goal-directed protocol, and warming devices were used to maintain normothermia. Prophylactic intravenous antibiotics were administered within 60 min before skin incision. Nasogastric tubes were not routinely placed, and pelvic drainage was used selectively.

Postoperative phase: A multimodal opioid-sparing analgesia regimen was applied uniformly, incorporating paracetamol and non-steroidal anti-inflammatory drugs (NSAIDs) as first-line agents, with opioids reserved for breakthrough pain. Early mobilization was encouraged according to a standardized schedule: patients were assisted to sit up within 24 h postoperatively, get out of bed within 48 h, and ambulate independently by day 3. Early oral feeding was initiated with clear liquids on the first postoperative day and advanced to a regular diet as tolerated. Intravenous fluids were discontinued early once oral intake was adequate. Urinary catheters were removed on postoperative day 1 or 2. Prophylactic cephalosporin antibiotics were administered within 24 h postoperatively.

### Surgical site infection (SSI) definition and surveillance

Surgical site infections (SSIs) were defined according to the criteria established by the U.S. Centers for Disease Control and Prevention (CDC), and were categorized into superficial incisional, deep incisional, and organ/space infections. All patients received standardized perioperative prophylactic antibiotic regimens, and preoperative mechanical bowel preparation combined with oral antibiotics was routinely administered. Postoperative surveillance was performed daily during hospitalization, and continued through scheduled outpatient follow-up visits and telephone interviews at 1, 3, and 6 months postoperatively. The diagnosis of SSI was confirmed independently by two attending physicians based on physical examination, laboratory findings, and wound culture results when indicated. Five patients who developed SSI all had a midline abdominal incision with an approximately 5-cm mini-laparotomy located above the umbilicus. In all cases, the infections were classified as deep incisional SSI involving the anterior rectus sheath. Wound care, including daily dressing changes, was provided, during which the wound status was closely monitored. The onset of infection was identified on postoperative days 4–5. All five cases of SSI were successfully managed with outpatient wound care and achieved complete healing without the need for reoperation.

### Surgical procedures

**Figure 1:**
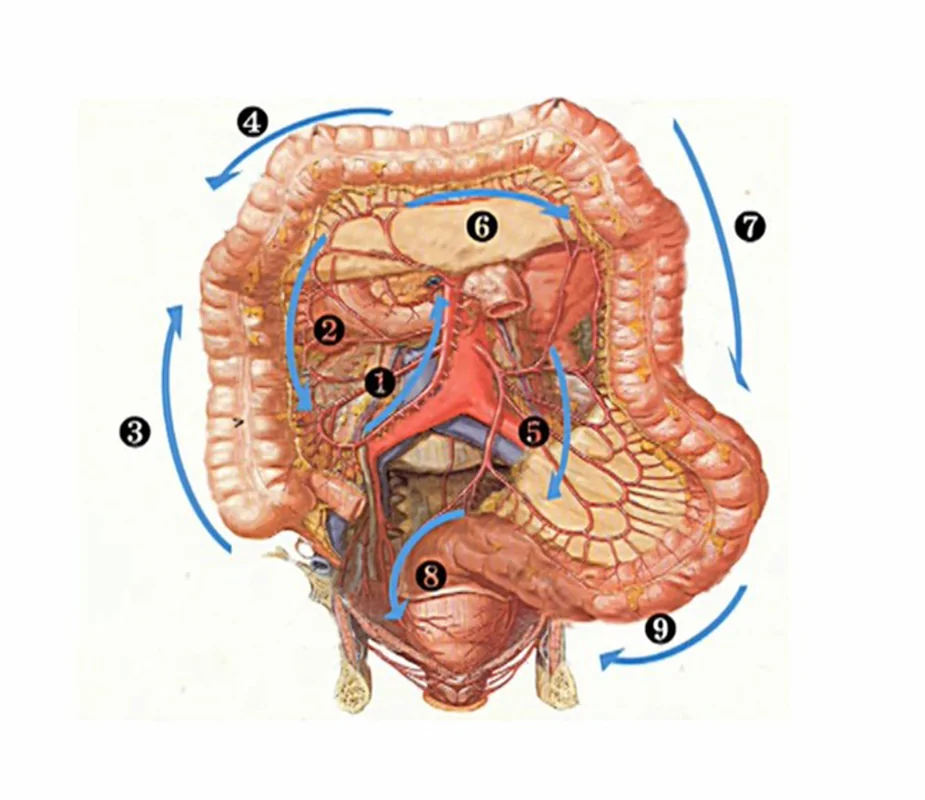
Sequential mobilization of the colon. Panels 1 to 9 illustrate the step-by-step operative procedure, which consists sequentially of mobilization of the right colon, left colon, sigmoid colon, and upper rectum.

All operations were performed by a single experienced team. Surgical Procedure for Laparoscopic-Assisted Natural Orifice Specimen Extraction (LA-NOSE) Subtotal Colectomy for Dolichocolon (Figure 1).

**Figure 2:**
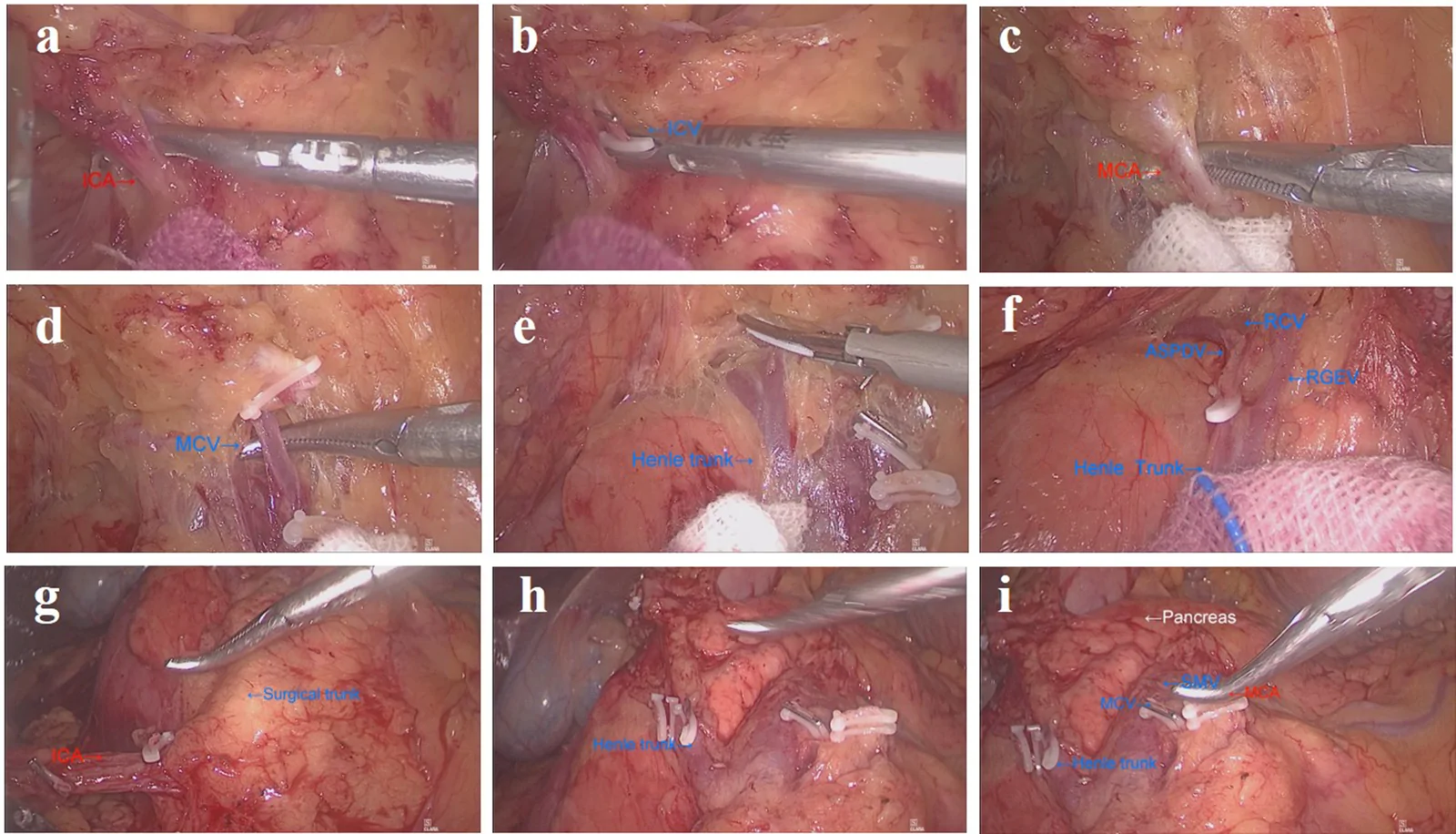
Right colon mobilization. Panels **(a–f)** depict the procedural sequence: sparing the ileocolic artery (ICA), transecting the ileocolic vein (ICV), transecting the middle colic vessels (MCA and MCV), and transecting the Henle trunk. Panels **(g–i)** display the vessels and surrounding tissues following right colon mobilization.

The first step is the mobilization of the right colon (Figure 2). The patient was placed in the supine split-leg position with a 15° Trendelenburg tilt and 10° left tilt. The main monitor was positioned at the right cranial side of the patient. The surgeon stood on the patient's left, the first assistant on the right, and the camera assistant between the legs. Mobilization of the right mesocolon was performed from caudal to cranial along the surgical trunk (superior mesenteric vein). The ileocolic vessels were identified, and the ileocolic vein was clipped and divided, whereas the ileocolic artery was preserved. Dissection continued proximally along the surgical trunk to isolate the right colic artery and the middle colic vessels, which were clipped at their origins with composite vascular clips and divided. The gastrocolic trunk of Henle was then dissected and divided approximately 0.5 cm from its confluence with the superior mesenteric vein using a composite vascular clip. Subsequently, the right mesocolon was mobilized medially to laterally within the Toldt's space, with meticulous protection of the descending duodenum and the uncinate process of the pancreatic head. The lateral peritoneal reflection of the ascending colon was incised from the cecum toward the hepatic flexure, and the hepatocolic ligament was transected, taking care to safeguard the right ureter and gonadal vessels. The gastrocolic ligament was opened to connect with the previously mobilized ascending mesocolon, completing the mobilization of the right colon.

**Figure 3:**
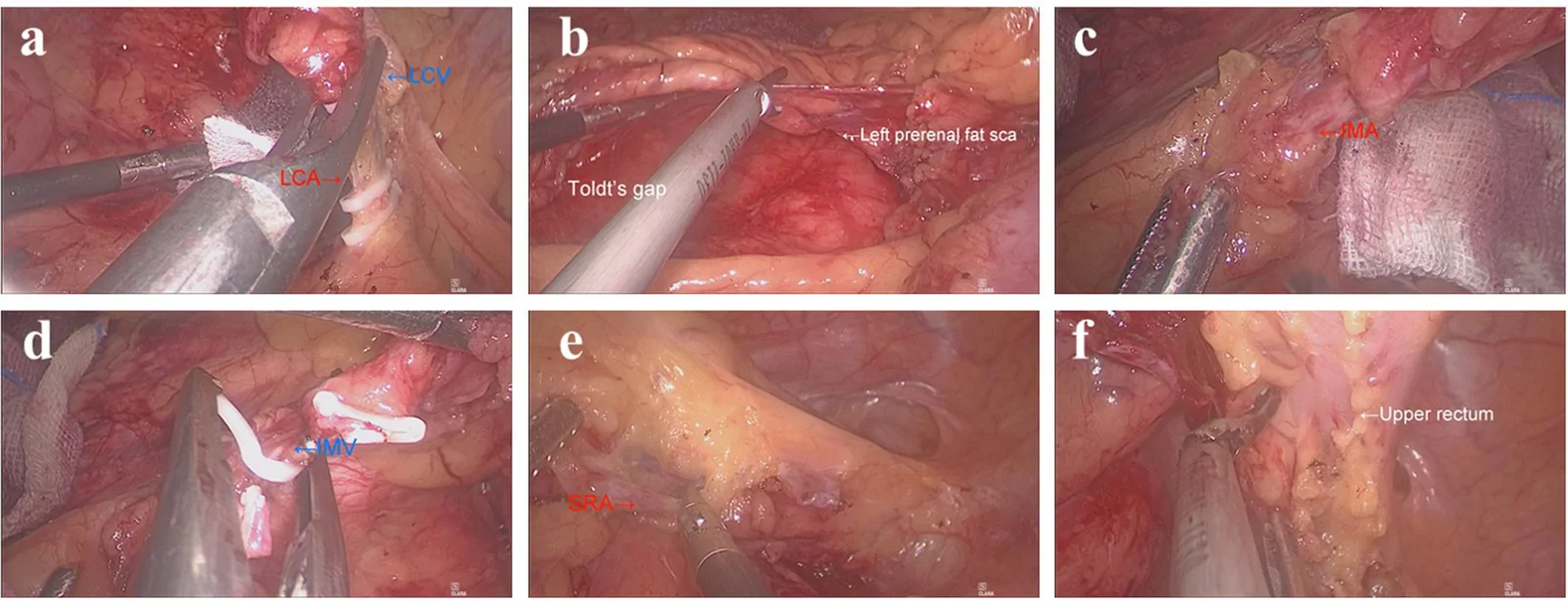
Left colon and rectal mobilization. Panels **(a–e)** illustrate the sequential vascular division steps: transection of the left colic artery and vein (LCA and LCV), transection of the inferior mesenteric artery and vein (IMA and IMV), and transection of the superior rectal artery and vein (SRA and SRV). Panel **(f)** shows the completed mobilization extending to the upper rectum.

The second step is the mobilization of the left colon (Figure 3). The patient was repositioned in the supine split-leg position with a 15° Trendelenburg tilt and 10° right tilt. The main monitor was moved to the left cranial side. The surgeon moved to the patient's right, the assistant stood between the legs, and the camera assistant took position to the surgeon's right. The left mesocolon was retracted, and the peritoneum overlying the descending mesocolon was incised to the right of the inferior mesenteric vein (IMV) to access the left Toldt's space. The IMV was clipped and divided at its root. Dissection was extended laterally within the Toldt's space, superiorly to the inferior border of the pancreas and the lower pole of the spleen, and laterally to the peritoneal reflection of the descending colon. The left colic vessels were identified and transected. The left transverse colon and the lateral attachments of the descending colon were sequentially mobilized, completing the mobilization of the left colon.

The third step is the mobilization of the sigmoid colon and the upper rectum. The patient was placed in the lithotomy position. The main monitor was relocated to the caudal end. The surgeon stood on the patient's right, the assistant on the left, and the camera assistant at the head side. The mesosigmoid and the mesorectum of the upper rectum were sequentially mobilized. At this point, the cecum, ascending colon, transverse colon, descending colon, sigmoid colon, and upper rectum were completely mobilized.

**Figure 4:**
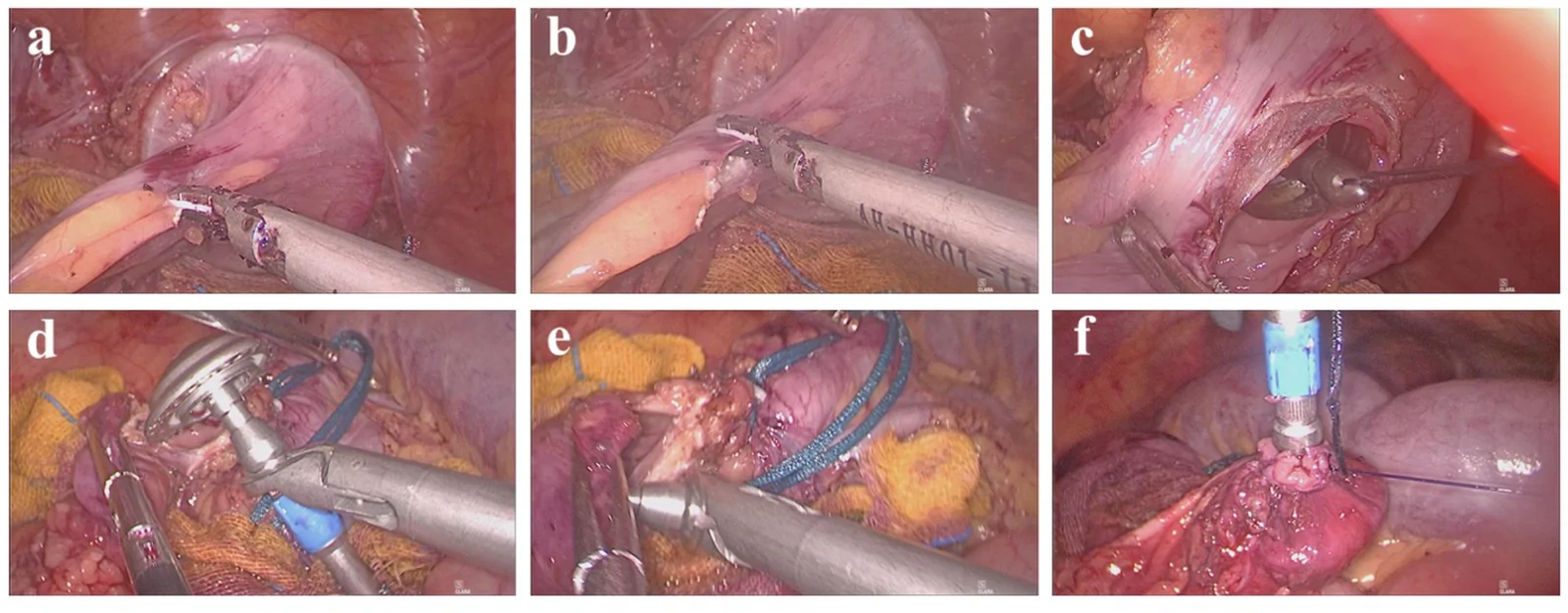
Transanal sleeve insertion and anvil placement. Panels **(a–f)** illustrate the sequential steps: insertion of a transanal sleeve, partial incision of the upper rectal wall, delivery of the circular stapler anvil through the sleeve into the pelvic cavity, and subsequent laparoscopic placement and fixation of the anvil into the ascending colon stump.

**Figure 5:**
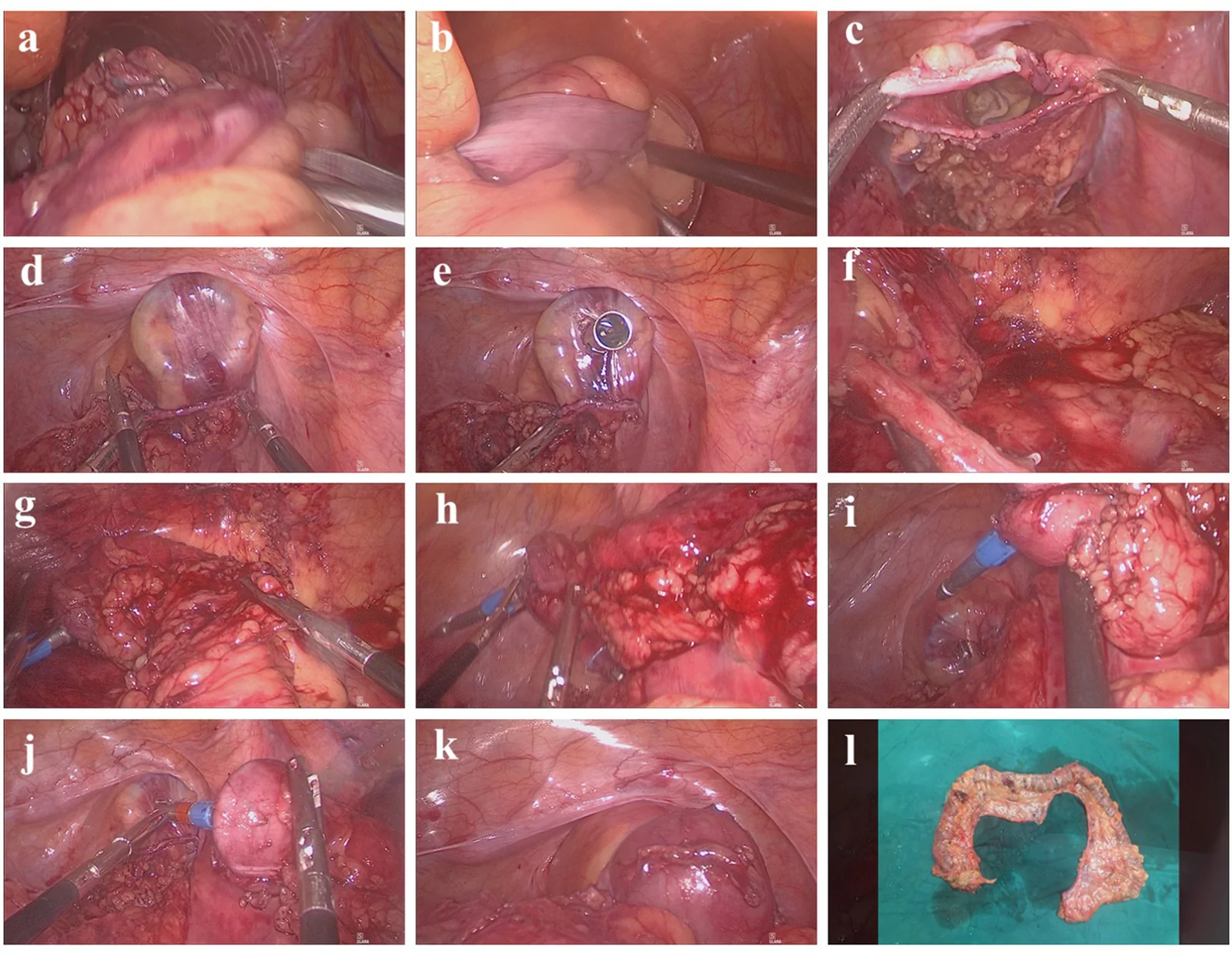
Transanal specimen extraction and anastomosis. Panels **(a–k)** illustrate the sequential steps: extraction of the resected colonic specimen through the transanal sleeve, closure of the upper rectal stump using an endoscopic linear stapler, counterclockwise rotation of the ascending colon, and performance of an end-to-end recto-ascending colon anastomosis. Panel **(l)** shows the entire resected colonic specimen.

The fourth step is the transanal retrieval of the specimen and anastomosis. The ascending colon was skeletonized approximately 5 cm from the ileocecal valve, and the upper rectum was skeletonized 3 cm above the peritoneal reflection. After antiseptic preparation, the anus was dilated to four fingerbreadths, and a self-constructed transanal sleeve was inserted. The rectum was transected full-thickness with an ultrasonic scalpel at the upper border of the skeletonized segment, and the anvil of a circular stapler was introduced into the pelvic cavity through the transanal sleeve, with meticulous care to protect the operative field and minimize contamination. The colonic wall was incised at the distal resection margin of the ascending colon, and the anvil with a pre-tied suture was placed into the proximal colonic lumen. An endoloop was introduced through a 12-mm port, snared around the tip of the anvil, and tightened to secure the anvil and close the proximal colon in preparation for anastomosis (Figure 4). The completely mobilized upper rectum, sigmoid colon, descending colon, transverse colon, ascending colon, and greater omentum were extracted *en bloc* through the self-constructed transanal sleeve. The rectum was divided with an endoscopic linear stapler approximately 1 cm distal to the transected edge. The resected rectal stump was placed in an endoscopic retrieval bag and removed through the 12-mm port. After removal of the transanal sleeve, the circular stapler was introduced transanally, and an end-to-end cecorectal anastomosis was created. The integrity of the doughnuts was confirmed. A pelvic drainage tube was placed, and the surgical field was irrigated with distilled water, completing the entire procedure (Figure 5).

All operations were performed by the same surgical team under general anesthesia. The colon was laparoscopically mobilized, with the ileocolic vascular pedicle and its branches preserved. After specimen extraction, the bowel was transected approximately 5 cm from the ileocecal junction, and the anvil of a 29-mm circular stapler was inserted into the distal ascending colon. The cecum was then placed into the pelvic cavity in a counter-clockwise fashion without torsion. Following appendectomy, a circular stapler was introduced transanally, and an iso-peristaltic cecorectal anastomosis was constructed between the cecum and the rectal stump, with special attention paid to achieve a tension-free anastomosis. The cecorectal anastomosis was subsequently reinforced with 3-0 barbed sutures. All mesenteric defects were closed to prevent postoperative internal hernia and colonic torsion.

Conventional laparoscopy-assisted subtotal colectomy: After laparoscopic mobilization and devascularization, the colon was transected. A mini-laparotomy (approximately 5–7 cm) was created in the abdominal wall to exteriorize the colon, resect the specimen, and insert the anvil of a circular stapler into the ascending colon stump. The stump was returned to the abdomen, and an end-to-end cecorectal anastomosis was completed transanally using the circular stapler.

**Figure 6:**
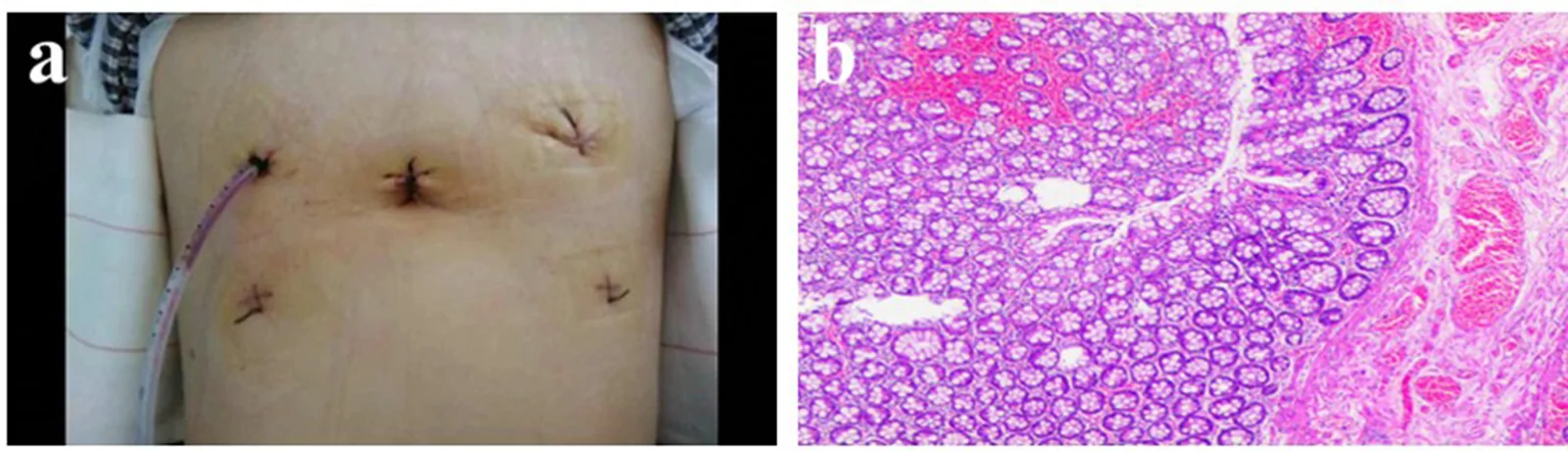
Postoperative abdominal appearance and pathological findings. Panel **(a)** shows the patient's abdominal wall appearance after surgery. Panel **(b)** presents the pathological diagnosis of the resected specimen (right colon, left colon, sigmoid colon, and upper rectum): chronic inflammation of the colorectal mucosa, with focal mucosal erosion, focal stromal vascular dilation, congestion and hemorrhage, and focal thinning of the bowel wall. In conjunction with the clinical presentation, these findings are consistent with a diagnosis of “redundant colon.”.

LA-NOSE subtotal colectomy: Laparoscopic subtotal colectomy was performed as above. Instead of an abdominal incision, a self-made tubular introducer was inserted transanally into the rectum. The anvil was delivered into the peritoneal cavity through this conduit and laparoscopically secured into the ascending colonic stump. The resected specimen was then extracted through the rectal conduit within a protective sleeve. After removal of the introducer, the circular stapler body was introduced per anum to fashion the cecorectal anastomosis. Thus, no abdominal extraction wound was required (Figure 6).

### Data collection and outcome measures

Data were extracted from medical charts, operative notes, nursing records, and a structured telephone follow-up (up to 8 months postoperatively). Baseline demographics included age, sex, and redundant colon classification (Types I–III) (8). Perioperative outcome measures comprised: (I) intraoperative parameters—operative time and estimated blood loss; (II) postoperative recovery milestones—time to sitting up, getting out of bed, unassisted ambulation, return of bowel function (flatus/stool), initiation of a liquid diet, and timing of suture removal; (III) complications—intestinal obstruction, anastomotic leakage, surgical site infection (SSI), and intra-abdominal infection.

### Statistical analysis

Categorical variables are presented as frequencies and percentages and were compared using two-tailed Fisher's exact test. Continuous variables (operative time and estimated blood loss) are presented as medians with interquartile ranges (IQR), as they were not normally distributed, and were compared between groups using the two-tailed Mann–Whitney *U*-test. Categorical variables are presented as frequencies and percentages and were compared using two-tailed Fisher's exact test. Statistical significance was set at *p* < 0.05. All analyses were performed using SPSS version 25.0 (IBM Corp., Armonk, NY, USA). The time thresholds for postoperative recovery milestones (e.g., sitting up within 24 h, out of bed within 2 days, ambulation within 3 days, bowel function return within 2 days, and liquid diet within 3 days) were defined *a priori* based on established ERAS guidelines and previous literature to ensure clinical relevance and avoid data-driven threshold selection (26). Statistical significance was set at *p* < 0.05. All analyses were performed using SPSS version 25.0 (IBM Corp., Armonk, NY, USA).

## Results

### Patient characteristics

This retrospective study included 34 consecutive patients who were diagnosed with chronic transit constipation (STC) caused by redundant colon at the Affiliated Hospital of Chengdu University between January 2009 and January 2026, and who underwent laparoscopic-assisted subtotal colectomy. Among them, 16 patients received laparoscopic-assisted natural orifice specimen extraction (LA-NOSE) surgery, while the remaining 18 underwent conventional surgery with specimen removal through a mini-laparotomy incision. The cohort comprised 25 females and 9 males, with a mean age of 45.2 ± 13.5 years in the LA-NOSE group and 46.8 ± 11.2 years in the conventional group (*p* = 0.67). According to the redundant colon classification, 8 patients were Type I, 22 Type II, and 4 Type III, with no significant imbalance between groups (Table 1). Baseline characteristics were thus comparable.

**Table 1:** Baseline demographics and disease characteristics.

Variable	LA-NOSE (*n* = 16)	Conventional (*n* = 18)	*P* value
Age (years), mean ± SD	45.2 ± 13.5	46.8 ± 11.2	0.67
Sex, F/M	12/4	13/5	1.00
BMI (kg/m^2^), mean ± SD	22.4 ± 2.1	22.8 ± 2.3	0.61
ASA grade, *n* (%)			0.738
I	6 (37.5)	8 (44.4)	
II	10 (62.5)	10 (55.6)	
Comorbidities, *n* (%)			
Hypertension	2 (12.5)	3 (16.7)	1.00
Diabetes mellitus	1 (6.3)	1 (5.6)	1.00
Coronary heart disease	0 (0)	1 (5.6)	1.00
Redundant colon type, *n* (%)			
Type I	4 (25.0)	4 (22.2)	1.00
Type II	10 (62.5)	12 (66.7)	1.00
Type III	2 (12.5)	2 (11.1)	1.00
Duration of constipation (years), median (IQR), All patients had a history of constipation >2 years.	5.0 (3.0–7.0)	5.5 (3.0–8.0)	0.52
Previous laxative use, *n* (%)	16 (100)	18 (100)	1.000
Bulking agents	16 (100)	18 (100)	
Probiotics	16 (100)	18 (100)	
Osmotic laxatives	16 (100)	18 (100)	
Stimulant laxatives	16 (100)	18 (100)	
Colonic transit time (hours), mean ± SD	86.5 ± 8.2	85.8 ± 7.9	0.79
All patients had colonic transit time >72 h.			
Defecography, *n* (%)	16 (100)	18 (100)	1.000
Normal	16 (100)	18 (100)	
Anorectal manometry, *n* (%)	16 (100)	18 (100)	1.000
Normal	16 (100)	18 (100)	
Balloon expulsion test, *n* (%)	16 (100)	18 (100)	1.000
Normal	16 (100)	18 (100)	
Pelvic floor electromyography, *n* (%)	16 (100)	18 (100)	1.000
Normal	16 (100)	18 (100)	
Endoanal ultrasound, *n* (%)	16 (100)	18 (100)	1.000
Normal	16 (100)	18 (100)	

### Intraoperative outcomes

The median operative time was 312 min (IQR, 281–346) in the LA-NOSE group and 295 min (IQR, 264–337) in the conventional group, with no statistically significant difference between the two groups (*p* = 0.31, Mann–Whitney *U*-test). The median estimated blood loss was 93 mL (IQR, 75–121) in the LA-NOSE group and 74 mL (IQR, 56–103) in the conventional group (*p* = 0.09, Mann–Whitney *U*-test).

### Postoperative recovery

The LA-NOSE group showed significantly faster recovery across all measured milestones compared with the conventional group (Table 2). Notably, 87.5% of LA-NOSE patients could sit up within 24 h, compared to only 38.9% in the conventional group (*p* = 0.005). Similarly, 93.8% vs. 55.6% got out of bed within 2 days (*p* = 0.019), and all LA-NOSE patients were ambulatory by day 3 vs. 72.2% (*p* = 0.046). Restoration of gastrointestinal function was achieved by day 2 in 62.5% of the LA-NOSE group (vs. 27.8%, *p* = 0.045). Initiation of liquid oral intake within 3 days was more frequent (87.5% vs. 44.4%, *p* = 0.013). A striking difference was observed in wound suture removal: all LA-NOSE patients had their tiny trocar-site sutures removed on days 6–7, whereas no patient in the conventional group met this endpoint due to the presence of a mini-laparotomy incision requiring longer healing (*p* < 0.001). The mean Postoperative length of stay (Postoperative LOS) was 5.5 days in the LA-NOSE group, compared with 9 days in the conventional group. Fourteen patients (87.5%) in the LA-NOSE group were discharged within 7 days after surgery, whereas no patient (0%) in the conventional group was discharged within this timeframe (87.5% vs. 0%, *p* < 0.001). Surgical site infection occurred in 0 of the patients who underwent LA-NOSE subtotal colectomy (0%), compared with 5 patients (27.8%) in the conventional laparoscopic-assisted subtotal colectomy group (0% vs. 27.8%, *p* < 0.046).

**Table 2:** Comparative perioperative outcomes.

Outcome	LA-NOSE (*n* = 16)	Conventional (*n* = 18)	*p* value
Operative time (min), median (IQR)	312 (281–346)	295 (264–337)	0.31
Estimated blood loss (mL), median (IQR)	93 (75–121)	74 (56–103)	0.09
Sitting up within 24 h, *n* (%)	14 (87.5)	7 (38.9)	**0**.**005**
Getting out of bed ≤2 days, *n* (%)	15 (93.8)	10 (55.6)	**0**.**019**
Ambulation ≤3 days, *n* (%)	16 (100)	13 (72.2)	**0**.**046**
Bowel function return ≤2 days, *n* (%)	10 (62.5)	5 (27.8)	**0**.**045**
Liquid diet ≤3 days, *n* (%)	14 (87.5)	8 (44.4)	**0**.**013**
Suture removal on days 6–7, *n* (%)	16 (100)	0 (0)	**<0**.**001**
Postoperative LOS, *n* (%)	14 (87.5)	0 (0)	**<0**.**001**
Surgical site infection, *n* (%)	0 (0)	5 (27.8)	**0**.**046**
Intestinal obstruction, *n* (%)	1 (6.3)	1 (5.6)	1.00
Readmission within 30 days, *n* (%)	2 (12.5)	3 (16.7)	1.00
Anastomotic leakage, *n* (%)	1 (6.3)	1 (5.6)	1.00
Intra-abdominal infection, *n* (%)	1 (6.3)	1 (5.6)	1.00
Repeat surgery, *n* (%)	0 (0)	0 (0)	1.00

Significant *p*-values are marked in bold.

### Complications

The incidence of SSI was significantly lower in the LA-NOSE group (0% vs. 27.8%, *p* = 0.046). The rates of intestinal obstruction (6.3% vs. 5.6%), Readmission (12.5% vs. 16.7%), anastomotic leakage (6.3% vs. 5.6%), and intra-abdominal infection (6.3% vs. 5.6%) were nearly identical between the two groups, with no significant difference (*p* = 1.00 for all).

## Discussion

NOSES (natural orifice specimen extraction surgery) refers to a series of surgical techniques in which intra-abdominal and intrapelvic procedures, including resection and reconstruction, are performed laparoscopically or robotically and the resected specimen is retrieved through a natural orifice (rectum, vagina, or oral cavity), thereby avoiding an additional abdominal wall incision (27). This concept was initially established in colorectal surgery, where the International Guideline on Natural Orifice Specimen Extraction Surgery (NOSES) for Colorectal Cancer (2023 version) has standardized 21 procedures with confirmed oncological safety and improved short-term recovery (28). In gastric cancer, the recently published International Guideline on NOSES for Gastric Cancer (2025 version) further defines nine procedures with transoral, transrectal, and transvaginal specimen extraction routes (27). Beyond these two fields, NOSES has been successfully extended to gynecological surgery through transvaginal specimen retrieval, urological surgery including transvaginal nephrectomy (29), and hepatobiliary and pancreatic surgery, in which total laparoscopic pancreaticoduodenectomy with natural-orifice specimen extraction has been achieved (30). Nevertheless, dedicated international guidelines are currently available only for colorectal and gastric NOSES, whereas applications in upper gastrointestinal, hepatobiliary and pancreatic, urological, and gynecological surgery remain at an earlier stage of development and are not yet supported by specialty-specific NOSES guidelines. Collectively, these findings indicate that NOSES is not limited to colorectal surgery but represents an evolving, multidisciplinary technical platform with broad application prospects (27).

The principal finding of this comparative study is that the LA-NOSE group was associated with more favorable outcomes in postoperative recovery and wound-related morbidity compared with the conventional laparoscopy group (31, 32). The elimination of an abdominal extraction incision translates directly into superior outcomes across several enhanced recovery domains and merits a thorough mechanistic and clinical contextualization.

### Reduction in surgical site infection

A clinically and statistically significant reduction in SSI (0% vs. 27.8%) was observed in the LA-NOSE group. Abdominal extraction incisions, especially when located in the midline or in proximity to a potential stoma site, are prone to contamination during specimen delivery. In NOSES, the specimen is extracted through the rectal conduit within a protective sleeve, physically sequestering the abdominal wall from direct contact with the enteric contents. This technical nuance is fundamental to infection prevention (33). The absence of SSI underscores the value of transrectal extraction under stringent aseptic technique. Nevertheless, this finding confirms that transrectal specimen retrieval, when performed with meticulous aseptic technique, does not introduce new sources of infection that would offset this advantage. Nevertheless, the risk of intraperitoneal contamination during transrectal specimen retrieval must not be underestimated. Our protocol incorporated meticulous preoperative mechanical bowel preparation with oral antibiotics, intraoperative rectal irrigation with dilute povidone-iodine, protective isolation of the operative field with iodine-soaked gauze, and copious peritoneal lavage. The fact that intra-abdominal infection rates were low and identical between groups (6.3% each) supports the safety of this approach, reaffirming that the transrectal route does not inherently increase the risk of deep organ-space infections when proper surgical discipline is exercised (21). It should be acknowledged that the complete absence of SSI in the LA-NOSE group is, to a substantial extent, inherent to the elimination of the abdominal extraction incision.

### Accelerated postoperative recovery within an ERAS framework

The LA-NOSE group exhibited significantly earlier mobilization—sitting up, getting out of bed, and independent ambulation—as well as faster return of gastrointestinal function and oral nutritional tolerance (21, 34). The avoidance of an abdominal wall incision attenuates somatic pain and the neuroendocrine stress response, which are well-recognized impediments to early mobilization (35, 36). Reduced incisional pain diminishes opioid analgesic requirements, thereby mitigating opioid-induced ileus and promoting gut motility (37). This aligns mechanistically with the pillars of enhanced recovery after surgery (ERAS), which emphasizes minimally invasive techniques, multimodal opioid-sparing analgesia, early enteral feeding, and proactive mobilization (38, 39). Furthermore, the 100% rate of suture removal on days 6–7 in the LA-NOSE group starkly contrasts with the conventional group, where the mini-laparotomy mandated longer wound care and delayed suture removal. This early resolution of wound management facilitates earlier hospital discharge and patient satisfaction, consistent with the psychological benefit of a “scarless” abdomen that reduces illness-related distress and fosters a sense of rapid normalcy (34).

### Safety assessment

Our results demonstrate that LA-NOSE does not compromise surgical safety. Rates of anastomotic leakage, intestinal obstruction, and intra-abdominal infection were low and comparable between the two groups, confirming that cecorectal reconstruction can be performed without jeopardizing the blood supply or tension of the anastomosis. The use of a self-made transanal introducer, as described, provides a cost-effective alternative to commercial platforms, potentially facilitating wider adoption in resource-constrained settings. When analyzed as continuous variables, operative time and estimated blood loss were comparable between the two groups: the median operative time was 312 min (IQR, 281–346) in the LA-NOSE group vs. 295 min (IQR, 264–337) in the conventional group (*p* = 0.31), and the median blood loss was 93 mL (IQR, 75–121) vs. 74 mL (IQR, 56–103) (*p* = 0.09). The slightly longer operative time and higher median blood loss observed in the LA-NOSE group may partly reflect the learning curve effect inherent to this technically demanding procedure, which requires advanced laparoscopic skills and transanal operative coordination. Although these trends did not reach statistical significance, they warrant further evaluation in larger cohorts. Larger cohort studies are warranted to further evaluate these technical parameters.

### Comparison with existing literature and study positioning

Our results corroborate the growing body of evidence supporting NOSES for left-sided colectomies and total mesorectal excision (33, 40–44). The extension of NOSES to subtotal colectomy for benign redundancy is less commonly reported, lending strength to our focused analysis. Although this study exclusively enrolled benign redundant colon patients, the findings prompt discussion regarding the applicability of LA-NOSE in malignant disease (45). In malignant conditions, concerns regarding tumor seeding, positive circumferential resection margins, and peritoneal dissemination currently limit the widespread adoption of NOSES colectomy (46). However, emerging data from specialized centers suggest that with rigorous specimen containment and careful patient selection, NOSES can meet oncological safety standards (47). Large-scale, multicenter validation is imperative before extending LA-NOSE to colorectal cancer.

### In summary

The present study demonstrates that LA-NOSE subtotal colectomy offers significant advantages in reducing surgical site infections and accelerating postoperative recovery, without increasing the risks of anastomotic leakage, ileus, or other complications, thereby confirming its safety and efficacy. Although the current evidence awaits validation by prospective, multicenter studies, LA-NOSE undoubtedly represents a more minimally invasive and patient-friendly surgical option for patients with benign redundant colon. This technique not only aligns with the principles of enhanced recovery after surgery and reduces healthcare resource utilization, but also holds potential health-economic benefits by lowering wound-related morbidity and shortening hospital stay. Therefore, we advocate for the active dissemination of LA-NOSE in benign colorectal diseases, provided that appropriate patient selection and rigorous technical training are ensured. Further technical refinements and standardized multicenter collaborations are encouraged to advance this procedure to a higher level of clinical practice.

### Limitations

We acknowledge several important limitations. First, Given the retrospective, non-randomized, single-center design and small sample size (*n* = 34), this study is inherently subject to selection bias, which limits the generalizability of our findings. The observed associations should not be interpreted as causal relationships. The trends toward longer operative times in the LA-NOSE group, although nonsignificant, warrant evaluation in larger cohorts to determine if this translates to clinically meaningful differences (48). Second, this study was restricted to benign disease; its safety and efficacy in patients with colorectal malignancy or extensive intra-abdominal adhesions remain unverified (27). Third, validated pain scores, precise opioid consumption data, cosmetic satisfaction scores, and long-term quality-of-life metrics were not systematically captured. Fourth, the definition of redundant colon types and the subtle variations in surgical technique should be standardized across centers. Fifth, the non-contemporaneous allocation of surgical periods (LA-NOSE cases performed after 2019 vs. conventional cases before 2019) introduces a potential time-related bias. While perioperative ERAS protocols were standardized across the entire study period to minimize this confounder, improvements in surgical experience and postoperative care over time cannot be entirely excluded. This is a key limitation of the retrospective design. Sixth, Although we supplemented baseline characteristics including BMI, ASA grade, comorbidities, and objective functional assessments, the retrospective nature of this study precluded collection of comprehensive nutritional status scores (e.g., PG-SGA, NRS-2002) for all patients, which may limit the completeness of the comparability assessment. Future prospective studies should incorporate validated nutritional screening tools as part of baseline evaluation. Seventh, several clinically relevant endpoints were not available in this retrospective cohort. Validated postoperative pain scores and precise opioid consumption data could not be systematically captured due to the variability in documentation across the long study period. These parameters are important for comprehensively evaluating the analgesic benefits of wound reduction, and their absence should be addressed in future prospective studies with standardized pain assessment protocols. Eighth, this study focused primarily on perioperative safety and short-term recovery outcomes, rather than long-term functional results of slow-transit constipation surgery. The comparative advantage of LA-NOSE over conventional laparoscopy in terms of wound-related morbidity and recovery does not necessarily translate to superior constipation relief, bowel function, or quality of life. However, our team has previously published a dedicated analysis of functional outcomes (including validated constipation scores, bowel frequency, diarrhea, continence, obstructed defecation symptoms, and GIQLI) following subtotal colectomy in this patient cohort, demonstrating satisfactory symptom control and quality-of-life improvement. Readers are referred to that publication for comprehensive functional assessment (24). The present study should be interpreted as complementary to that work, addressing the perioperative comparative aspects rather than duplicating the functional analysis.

### Future directions

Future prospective randomized controlled trials should incorporate patient-reported outcomes (including validated pain scores, cosmetic satisfaction, and quality-of-life measures) and extend follow-up to assess functional outcomes, sexual and urinary function, and delayed complications such as incisional hernia in the conventional group and anorectal dysfunction in the LA-NOSE group. In addition, a formal health-economic analysis comparing the net cost savings from reduced SSI and earlier discharge against the potential investment in specialized instrumentation and training would be valuable. Finally, standardization of redundant colon classification and surgical techniques across centers is essential to facilitate multicenter collaboration and robust evidence generation.

## Conclusion

For patients with slow-transit constipation secondary to redundant colon, laparoscopy-assisted subtotal colectomy with natural-orifice specimen extraction may offer relatively notable advantages compared with conventional laparoscopic surgery. LA-NOSE is linked to faster postoperative recovery across multiple evaluated recovery milestones and a markedly lower surgical-site infection rate, alongside potential cosmetic benefits (no standardized cosmetic-satisfaction assessment was conducted in this study), while operative risks may not be increased. This technique serves as a safe, technically feasible, patient-centered potential alternative for the surgical management of benign colonic redundancy. Multi-institutional prospective studies with extended follow-up are warranted to validate these observations and further explore the utility of LA-NOSE in a wider spectrum of colorectal diseases.

## Data Availability

The original contributions presented in the study are included in the article/Supplementary Material, further inquiries can be directed to the corresponding authors.
